# Mergers with Differentiated Products: Where Do We Stand?

**DOI:** 10.1007/s11151-021-09810-5

**Published:** 2021-02-09

**Authors:** Tommaso Valletti, Hans Zenger

**Affiliations:** 1 Imperial College Business School, Imperial College London, South Kensington Campus, London, SW7 2AZ U.K.; 2 European Commission, MADO 17/014, 1049 Brussels, Belgium

**Keywords:** Differentiated products, Mergers, Unilateral effects, L11, L13, L40, L41

## Abstract

On the occasion of the 10th anniversary of the 2010 U.S. Horizontal Merger Guidelines, this article provides an overview of the state of economic analysis of unilateral effects in mergers with differentiated products. Drawing on our experience with merger enforcement in Europe, we discuss both static and dynamic competition, with a special emphasis on the calibration of competitive effects. We also discuss the role of market shares and structural presumptions in differentiated product markets.

## Introduction

When the revised U.S. Horizontal Merger Guidelines were issued a decade ago, they provided a concise summary of the main economic principles and approaches for assessing the competitive effects of horizontal mergers.^1^ Rereading them today, it is remarkable how well they have aged. As was the case then, this is an instructive document for competition practitioners that distills the most important methods for assessing horizontal transactions.

Many aspects of the Guidelines that may have seemed controversial at the time are common ground in competition analysis today. E.g., the initially heated debate about diversion-based tools such as upward pricing pressure has largely subsided. In other areas, the Guidelines promoted analytical principles that anticipated what researchers would confirm in more formal analyses in the decade that followed. E.g., in the case of innovation competition, the Guidelines put an early focus on contestability and innovation diversion.

The main strength of the Guidelines, however, was not that they endorsed radically new concepts at the time. Many of the methods that they propose had existed for some time and had previously been applied in individual cases. For instance, the origins of upward pricing pressure can be traced back at least as far as Shapiro (1996), which had already contained the basic idea for what would later be termed “gross upward pricing pressure index” (GUPPI). Since around 2005, the UK authorities had started implementing Shapiro’s early formulas for illustrative price rises in UK merger cases.^2^ And also the European Commission’s 2004 Guidelines had emphasized the central importance of high diversion ratios and margins (the two main ingredients of upward pricing pressure) for assessing competitive effects.^3^

The main virtue of the 2010 Guidelines was instead to bring together a disparate panoply of approaches that had been used in isolated cases to create a unifying framework for implementation. As Shapiro (2010) put it, the Guidelines reflect the transition of merger analysis “from hedgehog to fox.” Whereas the hedgehog knows one big idea (market shares), the fox knows many different ideas: the variety of economic tools that are tailored to different market environments as described in the Guidelines.

In Europe, the publication of the U.S. Guidelines coincided with an ongoing shift of EU merger control towards a more economic approach. That shift had been initiated several years earlier following a number of court defeats in which the Court of First Instance had criticized the Commission’s (lack of) economic analysis.^4^ These judgments had led to the creation of a Chief Economist Team (in 2003), the adoption of a new Merger Regulation, and the publication of Horizontal Merger Guidelines (both in 2004).^5^

When the U.S. Guidelines were issued in 2010, this transition of EU merger control towards effects-based analysis was already well underway. Arguably, the publication of the U.S. Guidelines with their focus on economic methods facilitated that process. Perhaps this can be felt most noticeably in two areas: First, the Guidelines supported the calibration of competitive effects through their endorsement of price pressure tools. In the decade that followed, the Commission employed such quantifications in a significant number of cases, in particular in consumer goods and mobile telephony markets.^6^ Second, the Guidelines supported enforcement in the area of R&D competition, where they endorsed investigating innovation theories of harm. In recent years, the Commission has brought several such cases on the basis of economic mechanisms that were also described in the Guidelines.^7^

On the occasion of the 10th anniversary of the revised Horizontal Merger Guidelines, this article provides an overview of the state of economic analysis of unilateral effects in mergers with differentiated products. We discuss both static and dynamic competition, with a special emphasis on the calibration of competitive effects. Besides providing a comparative treatment of different economic methods, we also describe the practical enforcement experience with different approaches in EU merger control from our personal perspective.^8^$$^{\text {,}}$$^9^

The remainder of the article is organized as follows: Section 2 focuses on price competition and discusses the calibration of unilateral effects using diversion-based tools such as upward pricing pressure. Section 3 goes on to assess innovation competition. Section 4 discusses the role of market shares and structural presumptions in differentiated product markets. Section 5 concludes with an outlook.

## Price Competition

### Diversion Ratios

One of the most prominent contributions of the 2010 Guidelines was to de-emphasize the prior focus on market shares for assessing competitive effects in differentiated product markets. Instead, the Guidelines place closeness of substitution at the heart of unilateral effects analysis: “*The extent of direct competition between the products sold by the merging parties is central to the evaluation of unilateral price effects*” (§6.1).

While the 1992 Guidelines had also recognized closeness of substitution as a relevant factor in differentiated product markets, they had upheld market shares as the central element of analysis.^10^ However, it is well-known that market shares can be off the mark in trying to account for consumers’ heterogeneous switching patterns between differentiated products. When robust data are available, it is therefore more sensible to assess competitive overlaps directly via diversion ratios than to rely on market shares as an imperfect proxy. As noted by the Guidelines, “*Diversion ratios between products sold by one merging firm and products sold by the other merging firm can be very informative for assessing unilateral price effects, with higher diversion ratios indicating a greater likelihood of such effects*” (§6.1).

Obtaining an estimate of diversion is feasible in many, though certainly not all, significant mergers: e.g., through switching data, bidding data, customer surveys, event studies, or demand estimation. Their interpretation, however, can be intricate. Contrary to market shares, different diversion ratios cannot be directly compared with one another, since they refer to different groups of products. Moreover, diversion ratios between two merging parties cannot be sensibly aggregated into a “combined diversion ratio” (say, in a similar way that shares can be added into a combined market share).

In our experience, it can therefore be instructive to display diversion ratios between the merging parties as *implied market shares*.^11^ Implied market shares state how large merging parties’ market shares $S_{1}$ and $S_{2}$ *would have to be* to accurately reflect the observed diversion ratios between them. Concretely, suppose diversion ratios $D_{12}$ and $D_{21}$ were proportional to market shares. In that case $D_{12}=S_{2}/\left( 1-S_{1}\right)$ and $D_{21}=S_{1}/\left( 1-S_{2}\right)$. Solving this pair of equations for $S_{1}$ and $S_{2}$ then yields the hypothetical market shares that are consistent with the observed competitive interaction between the merging parties:^12^

1 $$\begin{aligned} S_{1}=\frac{D_{21}\left( 1-D_{12}\right) }{1-D_{12}D_{21}} \end{aligned}$$

Implied market shares can be thought of as the merging firms’ shares in the competitive space in which they actually compete. They are easier to compare than diversion ratios, since they refer to the same set of products. Moreover, they can be added to generate a one-dimensional measure of closeness of substitution between the merging firms relative to third parties. Since implied market shares are expressed in the same units as ordinary market shares, they are also more intuitive. Contrary to ordinary shares, however, they take account of the true degree of substitution between the merging products.^13^

Using (1) reveals that already seemingly moderate levels of diversion can be an indication of a considerable competitive overlap. E.g., defendants sometimes argue that diversion ratios between the merging parties of (say) one third are “low,” because the vast majority of substitution involves third parties. Yet, Eq. (1) shows that the combined market share consistent with diversion ratios of one third is $50\%$. As stated in the Guidelines, “*A merger may produce significant unilateral effects for a given product even though many more sales are diverted to products sold by non-merging firms than to products previously sold by the merger partner*” (§6.1). Accordingly, the belief that only mergers between “closest” competitors is likely to cause competition concerns is a misconception.^14^

### Price Pressure Tools

#### Upward Pricing Pressure

Diversion ratios provide a good indication of the relative closeness of substitution between merging companies. Yet, they do not capture the absolute intensity of competition faced by firms in the market: E.g., even if the merging firms are significant alternatives for their respective customers, anticompetitive effects may not arise if the competitive pressure that is exercised by other firms is sufficiently strong.

A more complete assessment, then, requires going one step further: The Guidelines pursue this through the concept of *upward pricing pressure * (UPP) (Farrell and Shapiro 2010a). “*In some cases, where sufficient information is available, the Agencies assess the value of diverted sales, which can serve as an indicator of the upward pricing pressure on the first product resulting from the merger* ” (§6.1). As we shall see, this approach captures both the relative closeness of the merging parties and the absolute intensity of competition they are exposed to.

At the heart of the UPP approach is the observation that competing to win sales exercises a negative externality on the prospective merger partner. Indeed, attracting new customers inflicts competitive harm on the other firm through the cannibalization of business. Post-transaction, merging firms will take this “cost of competing” into account and thus act less aggressively.^15^

The likelihood that a sale by firm 1 cannibalizes business of firm 2 is given by the diversion ratio $D_{12}$. To determine the expected competitive harm of a stolen sale for firm 2, this likelihood must be multiplied by the financial damage of a lost unit of output, which is price $P_{2}$ minus incremental cost $C_{2}$. From a post-merger perspective, firm 1’s opportunity cost of making additional sales is therefore given by the “value of diverted sales” from firm 2:

2 $$\begin{aligned} UPP_{1}=D_{12}\left( P_{2}-C_{2}\right) \end{aligned}$$

In antitrust practice, this opportunity cost is usually expressed as a percentage of price $P_{1}$, which is called the *gross upward pricing pressure index* (GUPPI) (Salop and Moresi 2009). Denoting the incremental margin of good 2 by $M_{2}=\left( P_{2}-C_{2}\right) /P_{2}$, the effective cost of competing induced by a merger is thus given by:

3 $$\begin{aligned} GUPPI_{1}=\frac{UPP_{1}}{P_{1}}=D_{12}M_{2}\frac{P_{2}}{P_{1}}\text {.} \end{aligned}$$

With equal prices, (3) simplifies to $D_{12}M_{2}$—a simple diagnostic for merger effects that had already been proposed by Shapiro (1996). Upward pricing pressure is therefore driven by two simple and intuitive parameters: the diversion ratio to the respective merger partner (a measure of the closeness of substitution) and the merger partner’s incremental margin (a measure of pricing power). Hence, GUPPIs not only capture *whom* firms compete with but also *how much*. Anti-competitive effects are more likely if both of these parameters are significant.

Since the merged entity will start internalizing the opportunity cost of cannibalized sales post-transaction, a merger can be thought of as imposing a “tax on competing” with respect to purchases of the merging firms’ products (Farrell and Shapiro 2010a). One might be tempted to conjecture that the size of this tax is given by (3). As it turns out, however, it is somewhat larger, since (3) reflects the pre-merger externality from firm 1 on firm 2. GUPPIs therefore only measure the “first round” tax that is imposed by a merger. They ignore subsequent feedback effects, as the price reactions of firms in the market are iteratively passed through into higher post-merger prices.^16^ The full tax-equivalent of a merger, then, is determined by *post-merger* GUPPIs, which are not observable pre-transaction. For this reason, GUPPIs are a conservative measure that understates the effective cost increase that a merger imposes on products of the merged entity.

#### Compensating Marginal Cost Reductions

An ingenious alternative for gauging the price pressure that is caused by a merger that accounts for feedback effects between the merging parties is due to Werden (1996). Rather than asking which pre-merger tax would lead to post-merger prices, Werden asks which post-merger subsidy would lead to pre-merger prices. The result is called *compensating marginal cost reductions* (CMCRs):^17^

4 $$\begin{aligned} CMCR_{1}=\frac{D_{12}M_{2}\frac{P_{2}}{P_{1}}+D_{12}D_{21}M_{1}}{ 1-D_{12}D_{21}} \end{aligned}$$

Consistent with the subsidy interpretation, CMCRs measure the size of marginal cost efficiencies that would be necessary to offset the upward pricing pressure that is caused by a merger. Expressed in those terms, CMCRs fully capture the unilateral effects of a transaction for arbitrary demand functions. Even so, the information required remains limited to diversion ratios and margins. Note, in particular, that (4) is independent of cost pass-through. This reflects the fact that both merger efficiencies and upward pricing pressure can be viewed as a cost change (the former a decrease, the latter an increase). When efficiencies and price pressure exactly offset each other, as they do in (4), it is therefore not necessary to know at which rate costs are passed through into final prices.^18^

As it turns out, CMCRs determine critical efficiencies not only for marginal cost savings but also for merger-induced improvements in product quality. Willig (2011) considers a model where mergers lead to an increase in product quality that is measured in terms of consumers’ willingness to pay for it.^19^ He uses a UPP framework to analyze post-merger incentives to raise quality-adjusted (hedonic) prices. In Annex 6.1, we show that the critical level of quality improvements that is required to avoid an increase in hedonic prices in the Willig model is simply given by (4). Hence, CMCRs measure critical efficiencies not only in terms of compensating marginal cost reductions, but also in terms of compensating (uniform) quality improvements.

Mathematically, CMCRs are an extension of GUPPIs that accounts for feedback effects between the merging firms’ prices.^20^ This can be seen in (4). The first term in the numerator is simply the $GUPPI_{1}$. The second term $D_{12}D_{21}M_{1}$ represents the first-round feedback effect from firm 2 (the $GUPPI_{1}$ of $GUPPI_{2}$). The denominator $1-D_{12}D_{21}$ represents the higher-order iterations of feedback effects between firms 1 and 2. Both of the added terms increase (4) relative to (3), so CMCRs are generally larger than GUPPIs – potentially significantly so when diversion ratios are high.

Since CMCRs are GUPPIs with feedback effects, one might be tempted to conjecture that they also measure the (full) tax on competing that is effectively created by a merger. However, in general this is not so. To see this, note that the pre-merger tax that generates post-merger prices is passed through at the pre-merger pass-through rate (which reflects a cost increase for two independent competitors). The post-merger subsidy that generates pre-merger prices is instead passed through at the post-merger pass-through rate (which reflects a cost decrease for a single merged entity).^21^ These pass-through rates will often differ for a given demand system, as they are based on market structures with different degrees of competition. As a result, CMCRs can be smaller or larger than the effective tax on competing that is created by a merger, and the difference can be significant.^22^ We illustrate this in Annex 6.2, which compares different price pressure tools in terms of predicted competitive effects. It is shown there that the ranking of price pressure indices crucially depends on the size of the cost pass-through.

#### Use of Price Pressure Tools

Price pressure tools such as GUPPIs and CMCRs have considerable advantages over more traditional forms of analysis. First, they accurately reflect both the intensity of competition in the market and the relative closeness of substitution between the merging parties. Second, they have limited informational requirements that can often be satisfied in significant mergers. Third, they do not require defining markets. Fourth, they allow the seamless integration of efficiencies into the competitive analysis. Finally, they have simple and intuitive interpretations: the pre-merger tax that would generate post-merger prices (GUPPIs) and the size of the efficiencies that would be needed to prevent competitive harm (CMCRs).

The European Commission has used price pressure tools on a significant number of occasions. Most frequently, GUPPIs and CMCRs were used in the assessment of mobile telephony mergers: e.g., the mobile mergers in Austria (2012), Ireland (2014), Germany (2014), Denmark (2015), the UK (2016), Italy (2016), and the Netherlands (2018).^23^ With the exception of the Dutch mobile decision, these cases ended either in remedies or in prohibition/abandonment (UK and Denmark).^24^

GUPPIs and CMCRs were used in these cases both in phase I and II alongside other quantitative methods.^25^ Conducting a second phase often allowed more precise estimates of the relevant inputs for calibration: E.g., the Commission typically used number portability and accounting data to determine diversion ratios and margins in phase I, whereas phase II often permitted conducting customer surveys and measuring incremental costs more specifically. These improved data were used not only for calibrating price pressure tools, however. Indeed, these decisions tended to focus more prominently on calibrated merger simulations (and sometimes demand estimation) and used GUPPIs and CMCRs mostly as complementary evidence.^26^

In other cases, such as *H3G Austria/Orange Austria*, * BASF/Bayer*, or *FCA/PSA*, the Commission used price pressure tools as the primary quantitative evidence, without complementing it with a fully-fledged merger simulation.^27^ In yet other cases, the Commission used price pressure tools as an initial screen, while the final decision relied on merger simulation alone. For instance, this was the case in * Unilever/Sara Lee* (which employed demand estimation with a nested logit model) and *Orange/Jazztel* (which applied a calibrated merger simulation).^28^ Finally, various Commission decisions also refer to high diversion ratios and margins as evidence of closeness of substitution and market power without explicitly calibrating competitive effects. This approach is consistent with the Commission’s Guidelines, which list high diversion ratios and margins as significant factors that make anticompetitive effects more likely.^29^

Note that a large proportion of mergers in Europe that concern national or local markets are handled by the 27 national competition authorities (NCAs) rather than the Commission. Hence, downstream mergers that involve final consumers—which tend to be particularly well-suited for price pressure analysis—are often not dealt with by the Commission. However, many NCAs have also readily embraced the use of price pressure tools. The UK agencies, in particular, had a pioneering role in applying diversion-based methods. Already from around 2005, UK authorities had used the closely related method of indicative price rises (of which more in the following section) to gauge the likely competitive effects of mergers. From 2010 onward, the UK agencies also started using GUPPIs in many cases—in particular in retail mergers (most recently in the widely discussed *Sainsbury/Asda* prohibition).^30^ The CMA’s 2017 retail mergers commentary notes that GUPPIs are now “*the most commonly used measure*” for quantifying competitive effects in such mergers in the UK.^31^

### Merger Simulation

Next, we turn to the question of how upward pricing pressure impacts prices. This is the realm of merger simulation, which entails the calibration of likely price effects through the use of more specific economic models. As noted in the Guidelines, “*Where sufficient data are available, the Agencies may construct economic models designed to quantify the unilateral price effects resulting from the merger*” ( §6.1). We consider the static effects of a merger here and therefore take product attributes as given. Note, however, that merging firms may sometimes have an incentive to re-arrange their product array post-merger, which may reduce incentives to raise prices (see Sect. 2.4 below).

There are two broad categories of merger simulation that are used in antitrust practice: calibrated merger simulations and simulations that are based on demand estimation. Calibrated merger simulations are closely related to price pressure tools, since they attempt to predict price changes based on simple pre-merger observables such as market shares, diversion ratios, and margins. Merger simulation based on demand estimation instead relies on an econometric estimation of the parameters of some model of competition. For this reason, demand estimation tends to be more involved due to its more demanding data requirements.

#### Calibrated Merger Simulation

As should be clear from the previous section, the price changes that are caused by a merger are determined by the extent to which price pressure is passed through into post-merger prices. To calibrate price effects in this spirit, Jaffe and Weyl (2013) have proposed the so-called * first-order approach *(FOA). It uses information that is local to the pre-merger equilibrium (on price pressure and pass-through rates) to conduct a linear approximation of the price effects of prospective mergers. Through Monte Carlo simulations, Miller et al. (2016) show that FOA calibrations tend to produce accurate predictions of price effects if the utilized local measures of pass-through are sufficiently precise.^32^ But unfortunately, the informational requirements for obtaining reliable estimates of the required pre-merger pass-through matrix are typically insurmountable in merger control practice.^33^

As is well known, pass-through is highly sensitive to demand curvature, with more convex demand exhibiting larger pass-through rates and hence more pronounced merger effects.^34^ In the absence of more detailed information on demand, calibrated merger simulation therefore requires making non-trivial assumptions about demand form. In merger control practice, the European Commission has often assumed linear demand for the calibration of price effects—particular in mobile mergers.^35^ This is a conservative approach, because linear demand tends to generate considerably smaller price effects than do most other standard demand forms.^36^ Consequently, empirical economists have often viewed linear demand as a lower bound for convexity.^37^

A particularly simple form of linear calibration is the so-called * indicative price rise* (IPR) methodology. IPRs assume that non-merging firms do not change prices post-merger (e.g., because they are part of a competitive fringe). Shapiro (1996) first derived a closed form solution for IPRs with linear demand for the special case of symmetric firms. Hausman et al. (2011) analyze the general asymmetric case. Assuming Slutsky symmetry, the predicted price effect $\Delta P_{1}/P_{1}$ of a merger is then given by

5 $$\begin{aligned} \frac{\Delta P_{1}}{P_{1}}=\frac{1}{2}\frac{D_{12}M_{2}\frac{P_{2}}{P_{1}} +D_{12}D_{21}M_{1}}{1-D_{12}D_{21}}\text {.} \end{aligned}$$

A simple comparison of (4) and (5) immediately shows that $\Delta P_{1}/P_{1}=CMCR_{1}/2$. This follows because the price effect of a merger equals the CMCR multiplied by the post-merger pass-through rate of the merged entity. Since third parties do not change prices in the IPR model, post-merger pass-through is simply given by the monopoly rate for linear demand, which equals one half.^38^

The UK competition authorities were early adopters of IPRs between 2005 and 2010 (then based on Shapiro’s results for symmetric firms).^39^ Most of the earlier UK decisions employed isoelastic rather than linear demand, however, which is discussed further below.

A drawback of linear IPRs is that they ignore feedback effects between the prices of the merging parties and the prices of outsiders. For this reason, the Commission has put more emphasis on full linear simulation in its case practice, which incorporates rivals’ reactions but does not require significant additional information.^40^ If we use Neurohr’s (2019) formulation, predicted price effects in the full linear model are given by

6 $$\begin{aligned} \frac{\Delta P}{P}=\nabla _{P}\left( C\right) \frac{\Delta C}{P}\text {.} \end{aligned}$$

Here, $\Delta P/P$ denotes the vector of price changes, $\nabla _{P}\left( C\right)$ is the post-merger pass-through matrix for linear demand (i.e., the Jacobian of prices with respect to marginal cost), and $\Delta C/P$ is the vector of CMCRs (with entries for non-merging firms given by zero). Similar to (5), (6) reflects the fact that price effects are determined by the pass-through of price pressure into final prices (here expressed as CMCRs passed through at the post-merger pass-through rate). Due to the inclusion of competitor reactions, the entries of $\Delta P/P$ are generally larger than the respective IPRs.^41^

While linear demand constitutes a reasonable lower bound for convexity, economists have often regarded isoelastic demand as a sensible upper bound. E.g., Shapiro (1996) used linear and isoleastic demand as focal points that define a sensible range of calibrated price effects. Also Hausman (2010) proposes the use of linear and isoelastic demand as convexity bounds absent more specific information about demand curvature.^42^

Shapiro (1996) first derived a closed-form solution for mergers with isoelastic demand for the special case of symmetric firms. In Annex 6.3, we analyze the general asymmetric case. If we assume Slutsky symmetry, the predicted price effects are then given by:

7 $$\begin{aligned} \frac{\Delta P_{1}}{P_{1}}=\frac{D_{12}M_{2}\frac{P_{2}}{P_{1}} +D_{12}D_{21}M_{1}}{1-D_{12}D_{21}-M_{1}-D_{12}M_{2}\frac{P_{2}}{P_{1}}} \end{aligned}$$

Note that (7) is independent of variables that pertain to third parties even though it constitutes a full merger simulation, not merely an IPR. This is due to the fact that prices are strategically neutral with isoelastic demand. Therefore, outsiders’ prices do not change in response to a merger.

As in the case of linear demand, price effects with isoelastic demand turn out to be a variation on CMCRs. In fact, (7) is identical to (4) except for two additional terms that are subtracted in the denominator. As a result, the predicted price effects are larger than CMCRs (and potentially significantly so when margins are appreciable). This reflects the fact that isoelastic demand has a pass-through rate that exceeds one.

Although isoelastic demand is often used by empirical researchers, we are not aware that it has been used for calibrated merger simulation by the Commission so far. However, the UK competition authorities have frequently assumed isoelastic demand in retail cases.^43^ Earlier UK decisions argued that this is the most plausible curvature assumption in retail markets.^44^ We would instead view isoelastic demand as an upper bound for possible merger effects – i.e., a worst-case scenario that can be used in conjunction with linear demand to indicate a possible range of price effects in the absence of more specific information about the shape of demand.

#### Merger Simulation Based on Demand Estimation

When sufficiently granular data are available, demand estimation can help overcome some of the limitations of calibrated simulation. In particular, merger simulations can then be based on an econometric estimation of the key parameters of competition – e.g., diversion ratios – that may not otherwise be available. Moreover, demand estimation also allows estimating more complex properties of demand. This may permit the use of less restrictive functional form assumptions – or at least to test statistically whether the underlying data are consistent with the imposed restrictions.

In this spirit, the academic literature has developed sophisticated structural estimation techniques.^45^ In merger control practice, however, structural estimation is often not feasible due to data, time and resource limitations. Moreover, where feasible, the robustness of structural models can be an issue that may limit their probative value, since the complexity of structural models can make them sensitive to changes in assumptions.^46^ Antitrust practitioners have therefore tended to favor less complex estimation techniques (e.g., nested logit models) over more complex ones (e.g., random coefficients logit models). Which specific model is best suited for demand estimation of course depends on the industry in question and on the availability of data in a given case.

The Commission has used demand estimation to inform the competitive analysis on a significant number of occasions, particularly in fast-moving consumer goods and mobile telephony markets. For instance, nested logit models were used in *TomTom/Tele Atlas*, *Kraft/Cadbury*, * Unilever/Sara Lee*, *Telefónica Deutschland/E-Plus*, and * TeliaSonera/Telenor*.^47^ See Buettner (2016) for a discussion (and references to additional cases that use merger simulation in different contexts).

### Comparative Analysis of Analytical Tools

The European Commission has used practically all of the tools that are discussed in this section, from implied market shares to demand estimation. Often different methods were used in the same decision. Other decisions mostly relied on one particular quantitative measure. This raises the question of whether different quantitative tools should be viewed as substitutes or as complements. Put differently: is there a natural hierarchy of methods that are preferable over others?

#### Relationship Between Diversion-Based Tools

**Fig. 1:**
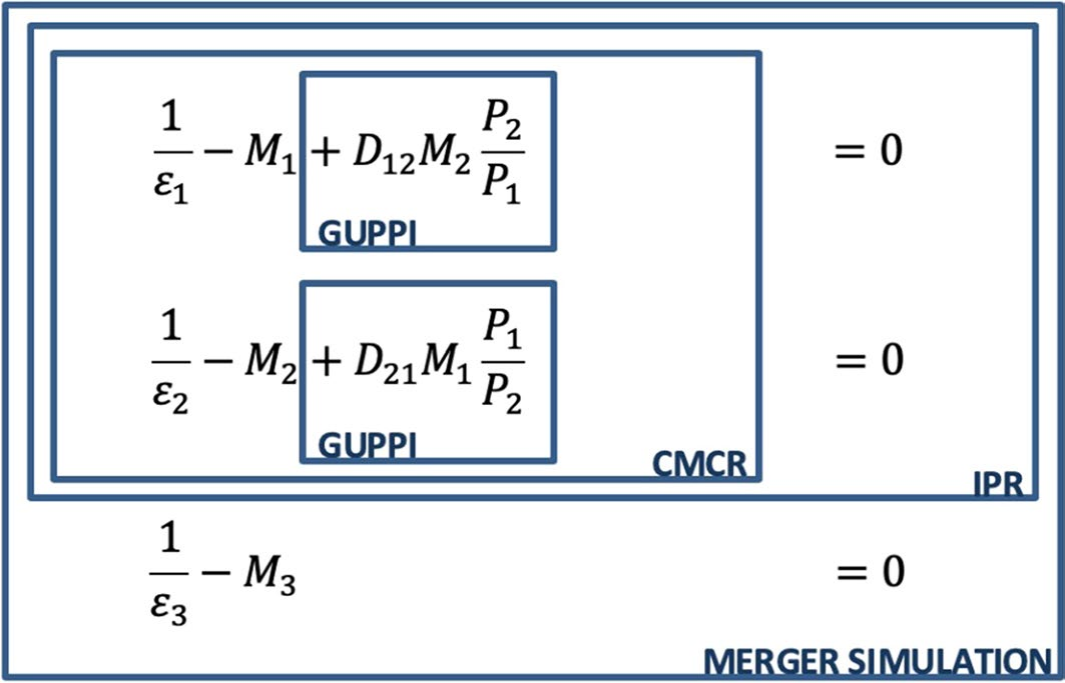
Post-Merger First-Order Conditions

Figure 1 illustrates the economic relationship between GUPPIs, CMCRs, IPRs, and merger simulation based on firms’ first-order conditions (FOCs) for profit maximization. In this system of equations, the GUPPI terms represent the change in pricing incentives in the merging firms’ FOCs compared to the pre-merger situation.^48^ As discussed in Sect. 2.2, GUPPIs measure the negative externality that a firm exerts on its merger partner, which will be internalized post-transaction. CMCRs go one step further by also taking account of the feedback effects between the merging firms’ FOCs. As a result, they are larger than GUPPIs. IPRs additionally account for the pass-through of price pressure into final prices (at the expense of having to assume a specific demand form). Finally, calibrated merger simulation also incorporates the feedback effects between the merging parties’ FOCs and those of outsiders. As a result, predicted price effects are larger than IPRs for a given demand system.

In using these tools, there is generally a trade-off between complexity and precision. More complex methods such as merger simulation can give more precise predictions if the underlying model is correctly specified. However, that cannot always be presumed. Simpler methods such as price pressure indices therefore have countervailing advantages in terms of robustness, as they restrict attention to the core of the anticompetitive effect.^49^ On balance, this suggests the use of different approaches as complementary evidence to obtain a fuller picture of the potential drivers of competitive effects.

But there is also some degree of substitutability between different measures: E.g., IPRs and linear merger simulations make the same assumptions about demand form and have similar data requirements. Even so, merger simulation is a more complete measure of price effects as it also accounts for competitor reactions. Similarly, CMCRs and GUPPIs have identical data requirements, and neither method requires an assumption on demand form. Yet, CMCRs are a more complete measure of price pressure, as they incorporate feedback effects between the merging firms.

GUPPIs have nonetheless proven to be a very useful tool in applied merger control. This is not only due to the intuitive appeal of viewing anticompetitive effects as a tax on the merging products, but also because GUPPIs turn out to be a sensible benchmark for conservatively estimating price effects. Indeed, Miller et al.’s (2017b) Monte Carlo simulations show that GUPPIs tend to be accurate predictors of price effects for demand functions with moderate convexity (e.g., linear or logit demand) and understate price effects for more convex demand forms (e.g., isoelastic or almost ideal demand).^50^

To understand this result, note that GUPPIs abstract from two factors as compared to a full merger simulation. First, GUPPIs ignore feedback effects between the merging firms, which leads to an underestimation of price effects. Second, GUPPIs ignore the rate of pass-through of price pressure into prices. This leads to an over- or underestimation of price effects, depending on whether pass-through is below or above one. When demand convexity is low, pass-through is below one. In that case the two factors that are omitted by GUPPIs go in opposite directions and approximately offset each other. When demand convexity is high instead, pass-through will be above one. In that case, both omitted factors lead GUPPIs to underestimate price effects. This is why GUPPIs can be viewed as a conservative estimate of price effects, which provides a useful lower benchmark in the absence of more specific information about the shape of demand.

By the same token, CMCRs can be viewed as an upper benchmark for conservatively calibrating price effects absent more specific information about demand curvature. This is equivalent to assuming that the merged entity’s pass-through rate is weakly smaller than one. For most purposes, this will be a cautious assumption as many common demand systems have pass-through rates in excess of one. Accordingly, Taragin and Loudermilk’s Taragin and Loudermilk (2019) Monte Carlo simulations find that also CMCRs often underestimate “true” price effects.

In sum, the use of GUPPIs as a lower benchmark and CMCRs as an upper benchmark provides a sensible conservative range for predicted price effects, which is simple yet instructive in the absence of more specific information about demand form.^51^ Moreover, recall that the “tax” and “subsidy” interpretations of these tools do not require making any assumptions about the shape of demand. This underlines the significant appeal of GUPPIs and CMCRs for merger control practice.

#### Application of Different Tools in European Case Practice

In recent European case practice, there has arguably been a focus on linear merger simulation, at the expense of the other methods that are described in this article. Although most of the decisions that were discussed in Sect. 2.3 also reported GUPPIs, CMCRs, and IPRs, the Commission’s emphasis was often on calibrated simulation. While the assumption of a particular demand form can be restrictive, the Commission noted that the specific assumption of linear demand “*is conservative as other forms of demand, such as log-linear demand, would imply a higher predicted price increase.*”^52^

Merging parties in those cases were hardly enthusiastic about simulations that predicted a potential for considerable price increases. Even so, the particular assumption of linear demand was typically not a central point of disagreement.^53^ Instead, merging parties tended to argue that the Commission’s estimates of diversion ratios and margins were excessive, or that static merger simulation fails to account for the dynamic benefits brought about by a transaction.^54^

This notwithstanding, the General Court in *CK Telecoms v. Commission *recently overturned the Commission’s prohibition decision in * Hutchison 3G UK/Telefónica UK *despite a prediction of considerable price effects.^55^ While the judgement does not reject the use of economic methods, it invokes a high standard of proof for the prohibition of mergers that do not lead to a dominant position. In particular, the Court held that the Commission must meet a higher standard of proof than the balance of probabilities in such cases. Moreover, it found that horizontal mergers should be judged against a presumption of standard efficiencies that are said to flow from such concentrations.^56^

Recent trends at the CMA appear to have been somewhat different than at the Commission. As was noted in Sect. 2.3, the UK authorities were early adopters of IPRs and built a strong reputation for conducting customer surveys (e.g., to estimate diversion ratios). In terms of instruments used, however, the CMA has tended to move away from IPRs towards GUPPIs.^57^ As we noted above, the CMA’s 2017 retail mergers commentary even describes GUPPIs as “*the most commonly used measure*” for the quantification of competitive effects.^58^

We have no strong views on whether a more simulation-centric approach (such as the Commission’s) or a more price pressure-centric approach (such as the CMA’s) is preferable. Both approaches are consistent with one another. What we would point out, however, is that consistency in the use of methodologies for similar cases can generate benefits in terms of comparability.

#### Limitations in the Use of Diversion-Based Tools

Finally, it should be recalled that diversion-based tools are designed to illuminate one specific aspect of competition: the change in static pricing incentives. While this is a crucial element of unilateral effects analysis, there can also be other—often dynamic—aspects of competition that may make adverse effects more or less likely: e.g., entry or expansion, potential competition, product repositioning, investments or efficiencies. In particular, adverse merger effects can only materialize if there is some form of barriers to entry.^59^

In some cases, the assumption of differentiated product markets with posted pricing that underlies the methods discussed in this article is not applicable. E.g., firms’ competitive position may not be due to product differentiation, but to some other market imperfection: e.g., capacity constraints or switching costs. Quantitative tools should therefore not be used mechanically, but in the context of a coherent theory of harm that combines quantitative with qualitative evidence, including an analysis of potential mitigating factors that may act as constraints on merger effects. E.g., in the mobile telephony mergers that were discussed above, the Commission often complemented its quantitative analyses with evidence from internal documents that pointed to “market repair” and “rational pricing” (euphemisms for higher prices) as firms’ primary goals in pursuing industry consolidation.^60^

## Innovation Competition

We now turn to innovation competition. Section 6.4 of the 2010 Guidelines is devoted to innovation and product variety and describes how the U.S. agencies evaluate horizontal mergers that may lessen innovation. “*The Agencies may consider whether a merger is likely to diminish innovation competition by encouraging the merged firm to curtail its innovative efforts below the level that would prevail in the absence of the merger. That curtailment of innovation could take the form of reduced incentive to continue with an existing product-development effort or reduced incentive to initiate development of new products.*” (§6.4)

This is consistent with the EC’s Horizontal Merger Guidelines, which also consider how a merger will impact innovation and, ultimately, consumers in the EU. The issue has gained considerable relevance in recent case enforcement in Europe—in particular in the agrochemical sector.^61^ The debate around mergers and innovation has also been much revamped recently amongst economists.^62^

### Theory of Mergers and Innovation

The earlier innovation literature (e.g., Aghion et al. 2005), which suggested a complex relation between innovation and competition, has often focused on issues such as innovation and market structure, which are related to but not identical to the question of how mergers affect innovation. Yet, it has become apparent that one cannot generalize the results of those papers easily to a merger context.^63^ Specifically, in that literature “more competition” is often modelled as a parameter that shifts the demand function of an innovator downwards.^64^ Instead, in a merger a fundamental channel that affects innovation incentives comes from the unilateral effects of a transaction: Firms also innovate *because* they want to drive demand away from competitors.

A more recent literature has therefore emerged that formalizes the main economic forces that are at play in mergers. These papers shy away from more general (but also generic) characterizations of innovation and competition and instead ask the more specific question of what happens in an industry before and after a merger when innovation is an important parameter of competition alongside other choice variables such as prices.

Perhaps the most important point that this literature has made has been to re-focus attention on the unilateral effects in innovation rivalry that may result from a merger. This is very much in line with the intuition that was developed in the 2010 Guidelines, which put an early focus on contestability and innovation diversion as a primary competition concern. E.g., §6.4 notes that a problem “*is most likely to occur if at least one of the merging firms is engaging in efforts to introduce new products that would capture substantial revenues from the other merging firm.*”

In this sense, the effect of a merger on innovation competition is very much like the diversion effect that is at the heart of the analysis of unilateral effects in price competition (Farrell and Shapiro 2010a). If additional investments in R&D by a firm reduce the expected profits of a rival (and vice versa) because innovation drives customers away, then a merger between the two firms internalizes this negative externality, which leads to less investment in R&D. While this is not the only effect of mergers on innovation competition, it is an important one that can give rise to competition concerns.

What is interesting about this more recent literature is the constant dialogue between economic research, application of the merger guidelines, and case practice. An early contribution was Motta and Tarantino (2018), who consider the effects of mergers on innovation with the use of different models of deterministic innovation. Concretely, they consider process innovation, so that higher expenditures lead to a certain reduction in the costs of production. In their main model, firms play a game where prices and investments are chosen simultaneously. In this setting results are clear. R&D investment follows quantities in the final product market; and since a merger typically increases prices and reduces output, R&D will fall as well. While outsiders react to this reduction in innovation by increasing their R&D and output, this is not enough to compensate for the merged entity’s reduction in investment. Motta and Tarantino (2018) also show that in the event that substantial efficiencies are achieved by a merger, the R&D result can be reversed.^65^

Federico et al. (2017, 2018) reach similar conclusions in a model of stochastic product innovation. Federico et al. (2017) consider a model where innovation is ex novo, and firms can eventually produce the same product if they successfully innovate.^66^ Federico et al. (2018) extend this framework to a setting where firms start with a baseline of existing differentiated products that can be improved by innovating. In both contributions, innovation is probabilistic in the sense that by spending more on R&D, a firm can improve its probability of achieving a successful innovation. Competing firms play a two-stage game, where price competition follows an initial stage of innovation competition. Therefore, the expected marginal gains from R&D in the first stage are driven by the difference between the expected profits with and without successful innovation in the second stage.^67^

Federico et al. (2017, 2018) show that, in the absence of efficiencies, the overall impact of a merger on innovation is determined by two effects: First, there is an “innovation externality” (or business stealing) effect, since increases in R&D expenditures by one firm reduce the expected profits of rivals (as discussed above).^68^ This negative externality on the merging partner will be internalized following a merger and thus lead to an unambiguous reduction in post-merger R&D efforts, all else equal.

Second, the merging firms will be able to coordinate the prices of the products in their different portfolios (including products where no innovation takes place). Federico et al. (2018) call this the “price coordination” effect. If the merger increases pre-innovation profits in the product market by more than it increases post-innovation profits, price coordination introduces a downward pressure on the merging firms’ incentive to innovate. If the converse is true, the merger exerts an upward pressure. Thus, in theory, this effect could go either way as far as innovation is concerned.^69^

Federico et al. (2018) use demand functions that imply that the price coordination effect is always positive, so that—in theory—it could be a proper countervailing force to the innovation externality.^70^ Since the model has technical complexities, Federico et al. (2018) resort to numerical parameterization. For the parameter ranges they consider, they find that the innovation externality generally prevails and outweighs the price coordination channel. While R&D investment by rivals increases, this is never enough to compensate for the loss that arises from the merged entity’s reduced investment incentives.

Similar to Motta and Tarantino (2018), in Federico et al. (2018) the innovation result can be reversed if there are sufficiently high merger-related efficiency gains, such as improvements in the effectiveness of innovation or reductions in R&D costs. Since a merger also reduces product market competition, however, post-merger prices always increase absent efficiencies. Hence, potential efficiency gains have to be quite strong to produce gains for consumers.

Denicolò and Polo Denicolò and Polo (2018) analyze one potential efficiency. Specifically, they show that in a variant of the model of Federico et al. (2017) a merger can lead to increased innovation if the convexity of firms’ R&D cost functions is sufficiently moderate. In that case, R&D coordination between the merging parties will lead to the shut-down of one of the firms’ R&D programs to avoid duplicative research. This concentration of R&D efforts can then lead to increased overall incentives to engage in R&D.^71^

This is an interesting point. However, we note some words of caution about the practical implications of the approach of Denicolò and Polo (2018): First, they mute price competition by assuming that all firms in the industry are colluding, so by assumption consumers cannot benefit from competition through lower prices. Second, they analyze only mergers to monopoly, and it is not clear how their result would generalize beyond this limiting case. This is an important point, as with more than two firms the R&D coordination effect for the merged entity would be significantly diluted. Third, they consider the impact of mergers only on innovation, while competition law is instead interested in the overall effect on consumers (including competitive benefits from lower prices). Finally, also in this model mergers soften R&D competition when no efficiency arises (which is the case when firms’ R&D cost functions are sufficiently convex).

Bourreau et al. (2018) investigate the impact of horizontal mergers on firms’ incentives to invest in demand-enhancing innovation in a more general framework. Their work is more directly related to Federico et al. (2018), although they consider a simultaneous move rather than a sequential move setting. They show that the results of Federico et al. (2018) generalize to a wide class of models that are customarily employed in the literature in Industrial Organization: the hedonic price model, quality-adjusted models, and CES demand. Results instead change when they introduce a “demand expansion effect”. This means that the R&D effort that is conducted by one firm would actually increase rather than decrease the demand for the products of its rivals. In this case, their setting is one with *positive* instead of negative R&D externalities among firms that, after the merger, get internalized. It is therefore not surprising that mergers can lead to more innovation in their model.

How can one summarize the findings from this recent debate among economists? While it is difficult to be unbiased, as one of the authors of this article is a co-author of articles that have recently caught the attention of the antitrust community, we believe that it is nevertheless possible to highlight the areas of agreement and disagreement.

First, it seems untenable to claim, as has been done by some commentators, that the prospect of higher prices alone following a merger will be sufficient to have a positive effect on innovation. This simply overlooks the fact that a primary motivation to engage in R&D is to attract customers from competitors.

Second, it seems equally untenable to argue that R&D—just because it is an uncertain process—cannot or should not be assessed. A variation of this claim is that authorities should look, for instance, only at pipelines in pharmaceutical mergers that are very likely to be developed into final products (the “D” leg in R&D), while one should stay away from basic research that is highly uncertain (the “R” leg in R&D). Essentially, this would be the equivalent of claiming that innovation competition should never be assessed—whatever the evidence.

Instead, we believe that there is no economic rationale for ignoring it. While competition authorities cannot advance speculative theories and have to meet the applicable burden of proof to the requisite standard, in some industries innovation is at the very core of the competitive interaction and should therefore be part of a regular merger assessment. The key concept that has to be understood is that authorities are not predicting winners, or successful products. Rather, they should assess how innovative efforts are used to compete (i.e., affect expected profits), and this can be sufficient to conduct a meaningful analysis.

Third, the economic framework has identified two main channels that capture the economic forces that are at play in the absence of efficiencies or uninternalized spillovers: the innovation externality channel (which always reduces innovation incentives) and the price coordination channel (which may reinforce or dilute the former). Therefore, to the extent that R&D expenditures spent today are geared at winning sales from rivals tomorrow (or protecting own sales from them), a merger between competitors with significant R&D overlaps can be problematic for competition.

Fourth, while innovation is important, it should not be forgotten that ultimately it is the impact on consumers that needs to be evaluated. The price coordination channel sometimes spurs innovation after a merger, as firms’ return on investment is increased with less competition. However, the same ex post increase in prices makes it less likely that consumers will be able to reap the benefits from innovation competition, as a good part of their surplus will be extracted by the merging parties. In other words, since higher future prices are bad for consumers (all else equal), the second channel will only rarely outweigh the first channel—unless there are strong efficiencies (such as R&D synergies) in addition.

Fifth, one should distinguish between situations where one firm’s R&D drives profits away from rivals and situations where innovation instead increases rivals’ profits. In practice, an important source of such pro-competitive complementarities can be knowledge spillovers. These are likely to play a greater role where the protection of IP rights is weak, or where licensing is highly imperfect.

More generally, efficiencies can improve merger effects if they are merger-specific. One such efficiency can be the ability to internally organize R&D more efficiently (as long as this cannot be achieved, say, by licensing). Forming a view on such potential benefits depends on a detailed knowledge of the R&D function. Hence, such analyses seem to fall naturally into the camp of the efficiency defense, for which the merging parties hold the burden of proof. Indeed, the evaluation of such claims depends on inside-the-industry knowledge that only the merging parties possess, who should therefore carry the burden of demonstrating such potential benefits to competition authorities.

Finally, there is no unique presumption that mergers are bad for innovation and consumers. The economics literature can therefore guide enforcers in their concrete policy choices. In view of the literature that has emerged in recent years, we conclude that it is reasonable for competition authorities to begin their analysis with the guiding principle that, in the absence of merger-related efficiencies—such as uninternalized positive externalities—a horizontal merger is unlikely to have positive effects on innovation incentives. Competition concerns are more likely to exist where innovation is a significant means of attracting customers from the merging partner (or, conversely, to protect existing business from it). Accordingly, authorities should not be receptive to the generic claim that mergers must be good for innovation—even in the absence of specific efficiencies—as this is an unlikely occurrence when R&D plays a significant role in the competitive rivalry between merging parties.

### Practice of Mergers and Innovation

While most of the discussion so far concentrated on the recent theoretical economic literature, considerable advances have also been made on the empirical front—despite computational challenges and data limitations in finding good proxies for innovation efforts. In particular, Igami and Uetake (2020) evaluate a structural dynamic oligopoly model that is applied to the hard disk drive industry. They find that mergers can be particularly harmful for innovation when an industry is already concentrated. Of course, these are the cases that matter in practice, as enforcers would not be likely to assess a merger in a fragmented industry with many competing players. Similarly, Cunningham et al. (2020) provide robust evidence of innovation concerns in the pharmaceutical industry. In their study of a large number of pharmaceutical acquisitions, incumbent firms often acquire innovative targets solely to discontinue the target’s innovation projects and thus pre-empt future competition. These studies confirm that innovation competition is often a central aspect of market interaction, which may be seriously derailed through a merger when innovation markets are concentrated to begin with.

Turning to case practice, the EU Horizontal Merger Guidelines point out that effective competition benefits consumers by promoting innovation and that mergers may deprive consumers of the value of improved and new products.^72^ Theories of harm that are based on innovation concerns have therefore been pursued by DG Competition in many instances.^73^ E.g., in the pharmaceutical and agrochemical industries, the Commission assessed overlaps between the merging firms’ pipelines (at various stages of development), their current products, and the assessed overlaps between lines of research.^74^ In engineering sectors—in particular in industrial gas turbines and in oil-field products—the Commission reviewed the R&D capabilities of the merging parties and their main competitors.^75^ In services areas, such as financial exchanges, the Commission analysed innovations that are related to products, technology, process, and market design.^76^

The case that generated the most attention recently is certainly * Dow/DuPont*.^77^ There, the Commission considered the possibility of increasing appropriability—the ability by an innovator to prevent knowledge spillovers to other firms—as a potential source of increased innovation incentives post-merger. However, the Commission found that such efficiencies were unlikely to play a significant role, since innovation in the crop-protection industry tends to be protected by effective IP rights. Appropriability was therefore already high prior to the merger. Concretely, most of the innovation in crop-protection takes place via the introduction of new products that are protected by effective patents. In successful instances, these products generate substantial sales with very high margins both during the patent period and after a patent expires.^78^ Therefore, there was no reason to restrict contestability between the merging firms to protect future rents. The investigation moreover showed that the markets for crop-protection products were already highly concentrated and had significant barriers to entry.

In this case, the Commission also engaged in a detailed analysis of the merging parties’ internal documents to identify the characteristics of research targets, R&D capabilities, and pipelines at the discovery (i.e., research) stage. The investigation showed that the merging parties had significant overlapping capabilities. Moreover, the Commission found direct evidence of a planned reduction of R&D capabilities compared to the situation without a merger.

In addition, the Commission carried out an analysis of patent data, which confirmed: (1) a high importance of both merging parties as innovators;^79^ (2) a high degree of concentration in research for new AIs at the discovery stage; and (3) a significant combined share of research for new AIs accounted for by the merging parties, notably in selective herbicides and insecticides.

The analysis of the patent data was also helpful to assess the degree of innovation closeness between the merging parties, and relied mainly on current lines of research, based on a review of the parties’ internal documents. In order to perform a similar exercise on past lines of research, the Commission identified the best-quality patents of the merging parties and requested the merging parties’ related documents (e.g., presentations and NPV calculations). This analysis revealed that the merging parties had also been close competitors for past innovations. Overall, the investigation showed that closeness in innovation was persistent, with the merging parties being close innovation competitors both for past innovations and for current innovations.

To address the Commission’s concerns on product market competition and innovation competition, Dow and DuPont offered to divest a large part of DuPont’s herbicide and insecticide businesses, as well as DuPont’s global R&D organization – including pipelines at the discovery stages for herbicides, insecticides, and fungicides, R&D facilities, and employees. This allowed the buyer of the divested businesses (FMC) to become a global and integrated R&D competitor.

## Concentration Measures in Differentiated Product Mergers

The 2010 Guidelines reflect the transition of merger analysis from the hedgehog of market shares to the fox of effects-based analysis, which takes account of multiple sources of evidence to engage in a substantive assessment. This raises the question of the role that concentration measures such as market shares and HHIs still have to play in differentiated product mergers. Views on this question could not be further apart. On one side of the spectrum, some observers have argued that concentration measures should be abandoned altogether in differentiated product mergers, to be replaced by an unrestrained analysis of competitive effects.^80^ Other commentators have shown far more unease with such substantive analyses and regard market shares as the determinative criterion for merger assessments.

In our mind, following either extreme would be a mistake. As this article has illustrated, diversion-based methods have considerable advantages over concentration-based measures in differentiated product markets, since they reflect closeness of substitution and market power much more accurately than market shares could ever hope to. Moreover, properly delineating markets requires essentially the same type of information that is also used to analyze competitive effects—in particular diversion ratios and margins.^81^ Yet, market definition produces only a crude “in or out” decision with these inputs, whereas diversion-based tools capture the underlying substitution patterns and competitive intensity that are contained in the data. This is why effects-based methods have considerably advanced the assessment of mergers with differentiated products.^82^

Even so, we believe that market shares rightfully continue to play an appreciable role in the analysis of differentiated product mergers. This includes the use of safe harbors for low-market-share mergers and rebuttable presumptions for high-market-share mergers that shift the burden of proof to the notifying parties. In our view, competition authorities ought to present particularly compelling effects-based evidence if they intend to raise competition concerns in markets with relatively lower market shares. Conversely, merging parties ought to present particularly compelling effects-based evidence if they want to dispel competition concerns in markets with relatively high market shares.

Of course a direct assessment of competitive effects will tend to be preferable to mere concentration analysis when the available data are robust. In that case market delineation can be tailored to fit the facts of a case, and market shares will inherently carry less weight. However, in a world of imperfect information, market data are certainly not always perfect. For instance, in many mergers switching data to measure diversion ratios are not available and where they are, they are not always reliable.^83^ In contrast, the sales data that are used for market share calculations tend to be widely available and highly reliable due to firms’ reporting obligations under the tax laws. The advantage of market shares is therefore the robustness of measurement, whereas the advantage of diversion ratios is the robustness of the measure.^84^ This militates for using both types of evidence in an investigation on their respective merits.

Of course, in situations where market data are imperfect, this may equally taint the delineation of market boundaries. However, that does not necessarily invalidate the use of market shares. In some cases, market shares may be problematic even in the widest plausible segmentation. In others, shares may be innocuous even in the narrowest plausible market. In yet other cases, market delineation may not be controversial at all. In any event, market delineation does not exclusively rely on quantitative data. Instead, also various types of qualitative evidence tend to inform market definition—especially in situations where the availability or reliability of quantitative evidence is unclear. Market share data can therefore provide useful information about the competitive positioning of different firms when more direct evidence is ambiguous or hard to come by.

Contrary to what is sometimes claimed, structural presumptions for high market share mergers are also well-grounded in economic theory.^85^ Virtually all standard models of Industrial Organization suggest that—in the absence of efficiencies—concentration by merger tends to be associated with increased prices and decreased consumer welfare. In some cases, this link between market shares and market power can be quite direct.^86^ A stricter approach toward acquisitions of firms with high market shares therefore rightly protects the more limited level of competition that still exists in highly concentrated markets.

Moreover, structural presumptions go some way towards addressing more complex competition concerns, such as impediments to potential competition, innovation competition, or coordinated effects. Such dynamic aspects of competition are particularly relevant in industries where, already prior to a proposed merger, firms exhibit substantial market power. In our previous work (Valletti and Zenger 2018, 2019) we discuss the structural increase in market power that many industries have experienced over the past 30 years according to the empirical literature. As we illustrate there, it is rational for competition authorities to apply comparatively stricter concentration benchmarks when pricing power is already substantial. This reduces the risk of type II errors, which are particularly costly for society when market power is high.^87^

Of course, none of this implies that market shares are a more desirable diagnostic tool than effects-based methods. As noted in Sect. 2, concentration measures can be misleading when substitution is not proportional to market shares. This is why economic methods that measure competitive overlaps and market power more directly have greatly improved merger analysis. Our point here is instead that high (low) market shares establish a plausible prior for (against) competition concerns absent more specific information about the likely effects of a merger. In this way, concentration analysis can play an important role in shaping the standard of proof against which effects-based analysis can be judged. As Lord Hoffman noted in *Rehman*, “*it would require more convincing evidence to conclude that it was more likely than not that the sighting of an animal in a park was a lion than it would be to satisfy the same standard of probability that the animal was a dog.*”^88^ In that sense, a continued role for market shares as a material piece of evidence is simply a matter of Bayesian inference.

## Outlook

Ten years after the revised U.S. Guidelines were issued, merger control is at an important juncture. According to the empirical literature, market power in oligopolistic markets has risen over recent decades in both the U.S. and the EU. E.g., the widely cited studies by De Loecker et al. (2020) and De Loecker and Eeckhout (2020) show that we are in the midst of a sharp increase in pricing power across the economy. This secular shift is in large part driven by a reallocation of economic activity towards larger, more profitable oligopolists. At the same time, aggregate investment has been falling, empirical measures of barriers to entry have risen, and productivity growth is in decline.^89^

In such an environment, vigilant merger control is particularly important to protect the functioning of the competitive process. As the empirical literature has shown, horizontal mergers can cause substantial competitive harm in oligopolistic markets. E.g., Ashenfelter et al.’s (2014, p. S78) survey of empirical merger retrospectives notes, “*The empirical evidence that mergers can cause significant increases in price is overwhelming. Of the 49 studies surveyed, 36 find evidence of merger-induced price increases.*” Thus, it is important to identify effective tools that can detect potentially anticompetitive mergers and distinguish them from benign transactions.

The U.S. Horizontal Guidelines set out a useful framework to do this. In this article, we have provided a comparative treatment of different economic methods that assess competitive effects in the spirit of the Guidelines. These tools draw on the key factors that determine competitive outcomes—principally: *who* do merging firms compete with, and *how much*—to assess whether or not a transaction is likely to lead to material unilateral effects. Such methods can be particularly helpful in non-dominance cases, where inferences from market shares alone are often not sufficient to distinguish between harmful and harmless transactions. Being able to enforce competition law in such cases is critical, however, because according to the empirical literature, many of the most problematic transactions occur in oligopoly markets.^90^

It is also important to recognize, however, that economic tools are not a panacea. In particular, quantitative predictions should not be interpreted as surgical point-estimates, but as directional measures that reflect the expected strength of underlying incentives.^91^ In our view, the U.S. Guidelines strike the right tone here, by stating that higher diversion ratios indicate “*a greater likelihood*” of unilateral effects (§6.1) (much as higher market shares indicate a greater likelihood of unilateral effects when firms are similarly positioned). Appropriately applied, quantitative techniques should therefore be embedded in a broader case that is based on a combination of qualitative and quantitative pieces of evidence to understand the likely competitive implications of a given transaction.

## Appendix

### Compensating (Uniform) Quality Increases

Consider that the merging firms 1 and 2 have demand $X_{1}$ and $X_{2}$, respectively. Following Willig (2011), assume that post-merger the quality of firm 1 and 2’s products uniformly increases (in terms of consumers’ willingness to pay for quality) by $V_{1}$ and $V_{2}$, respectively. In order to focus on these quality changes, we assume that marginal costs remain unchanged by the merger. Since the quality increases of goods 1 and 2 uniformly affect consumers, they correspond to a shifting out of the respective demand curves by $V_{1}$ and $V_{2}$, so post-merger demands are given by $X_{i}\left( P_{i}-V_{i},P_{j}-V_{j}\right)$ for firms *i* and *j* . We can thus work with hedonic prices, which are given by $\hat{P} _{i}=P_{i}-V_{i}$ for $i=1,2$. The merged entity’s post-merger profits are then given by:

$$\begin{aligned} \Pi =X_{1}\left( \hat{P}_{1},\hat{P}_{2}\right) \left( \hat{P} _{1}+V_{1}-C_{1}\right) +X_{2}\left( \hat{P}_{2}+V_{2}-C_{2}\right) \end{aligned}$$

Maximizing this expression with respect to $\hat{P}_{1}$ and rearranging yields

$$\begin{aligned} \frac{\hat{P}_{1}-C_{1}}{\hat{P}_{1}}-\frac{1}{\hat{\varepsilon }_{1}}+\frac{ V_{1}}{\hat{P}_{1}}-\hat{D}_{12}\frac{\hat{P}_{2}+V_{2}-C_{2}}{\hat{P}_{1}}=0 \end{aligned}$$

where variables with a hat denote measures using hedonic prices (e.g., $\hat{ \varepsilon }_{1}$ denotes the elasticity of demand for good 1 with respect to $\hat{P}_{1}$).

Now consider the case where $V_{1}$ and $V_{2}$ are at a level such that there is no post-merger increase in hedonic prices (i.e., $\hat{P} _{i}=P_{i}^{0}$ for $i,j=1,2$, where $P_{i}^{0}$ denotes the pre-merger price). Then, from the pre-merger first-order condition for product 1, $\left( \hat{P}_{1}-C_{1}\right) /\hat{P}_{1}=1/\hat{\varepsilon }_{1}$ and so

$$\begin{aligned} V_{1}=\hat{D}_{12}\left( \hat{P}_{2}+V_{2}-C_{2}\right) \end{aligned}$$

(and similarly for good 2).

Solving this system of equations for $\hat{P}_{1}$ and rearranging then yields

$$\begin{aligned} \frac{V_{1}}{\hat{P}_{1}}=\frac{\hat{D}_{12}\hat{M}_{2}\frac{\hat{P}_{2}}{ \hat{P}_{1}}+\hat{D}_{12}\hat{D}_{21}\hat{M}_{1}}{1-\hat{D}_{12}\hat{D}_{21}} \end{aligned}$$

(and similarly for firm 2). Noting that, by assumption, post-merger hedonic prices are equal to pre-merger nominal prices, this expression is identical to (5).

### Comparison of Price Pressure Tools

#### Comparison of Price Pressure Tools with Price Effects

We first consider the quantitative relation between three measures of upward pricing pressure: GUPPIs, CMCRs, and the effective tax on competition (TOC) that is caused by a merger. We then compare these indices with the “actual” price effect $\Delta P/P$.

For simplicity, assume that firms are symmetric and that the diversion ratio *D* from one firm to another is (approximately) constant over the relevant range.^92^ In that case, GUPPIs are given by *DM* where *M* denotes the incremental margin. Similarly, CMCRs are given by $DM/(1-D)$. If we denote the pre-merger pass-through rate of the merging firms by $\rho$, TOCs are defined as:^93^

8 $$\begin{aligned} \frac{\Delta P}{P}=\rho \cdot TOC \end{aligned}$$

If we denote the post-merger pass-through rate by $\rho ^{*}$, CMCRs can similarly be stated as:^94^

9 $$\begin{aligned} \frac{\Delta P}{P}=\rho ^{*}\cdot CMCR \end{aligned}$$

Note that TOCs are post-merger UPPs that are normalized by pre-merger prices. Since *D* does not change post-merger, TOCs can therefore alternatively be expressed as follows:

$$\begin{aligned} TOC=D\frac{P^{*}-C}{P} \end{aligned}$$

When we use this expression, (8) can be restated as

$$\begin{aligned} \frac{P^{*}-P}{P}=\rho D\frac{P^{*}-C}{P} \end{aligned}$$

which is equivalent to

$$\begin{aligned} \frac{\Delta P}{P}=\frac{\rho DM}{1-\rho D}\text {.} \end{aligned}$$

If we use (8), we therefore have

$$\begin{aligned} TOC=\frac{DM}{1-\rho D}\text {.} \end{aligned}$$

When we compare the various upward pricing measures, we then find the following. For low pass-through rates ($\rho \le 1/\left( D+1\right)$):

$$\begin{aligned} \frac{\Delta P}{P}\le GUPPI<TOC<CMCR \end{aligned}$$

For moderate pass-through rates ($\rho \in \left( 1/\left( D+1\right) ,1\right)$):

$$\begin{aligned} GUPPI<\frac{\Delta P}{P}<TOC<CMCR \end{aligned}$$

For high pass-through rates ($\rho \ge 1$):

$$\begin{aligned} GUPPI<CMCR\le TOC\le \frac{\Delta P}{P}\text {.} \end{aligned}$$

In essence: When pre-merger pass-through equals one ($\rho =1$), CMCRs and TOCs are identical, and both are exact measures of the price effects of a merger, whereas GUPPIs underestimate price effects. When demand functions are more convex ($\rho >1$), all upward pricing measures understate the actual price increase. Conversely, when demand functions are less convex ($\rho <1$), most upward pricing measures overstate the actual price increase, with the exception of GUPPIs, which may over- or understate them, depending on the precise level of pass-through. Hence, GUPPIs tend to approximate price effects well for demand with moderate convexity but tend to understate them for more convex demand.

#### Comparison of Price Pressure Tools with Calibrated Merger Simulation

Next, we compare the size of predicted price effects from calibrated merger simulation models with the size of price pressure tools. In Sect. 2.3, we have already shown that for a given set of parameters linear IPRs are smaller than CMCRs, which in turn are smaller than the price effects with isoelastic demand.

It remains to be shown how GUPPIs compare to IPRs. With the assumption of symmetry, GUPPIs are again given by *DM*, whereas IPRs are given by $DM/2(1-D)$. Hence, GUPPIs are larger than IPRs if and only if $D\le 1/2$, which in a symmetric model holds for all mergers except mergers to monopoly. As long as there are at least two firms post-merger, we therefore have

$$\begin{aligned} IPR<GUPPI<CMCR<IMS \end{aligned}$$

where *IMS* denotes isoelastic merger simulation.

### Calibrated Merger Simulation with Isoelastic Demand

Assume isoelastic demand. Firms 1 and 2 have demand $X_{1}$ and $X_{2}$, respectively. Pre-merger, firm 1 maximizes profit $X_{1}\left( P_{1}-C_{1}\right)$. This leads to the Lerner equation $M_{1}=1/\varepsilon _{1}$ where $\varepsilon _{1}$ denotes the elasticity of demand of firm 1.

Post-merger, the merged entity maximizes profit $X_{1}\left( P_{1}-C_{1}\right) +X_{2}\left( P_{2}-C_{2}\right)$. This leads to:

10 $$\begin{aligned} M_{1}^{*}=\frac{1}{\varepsilon _{1}}+D_{12}^{*}M_{2}^{*}\frac{ P_{2}^{*}}{P_{1}^{*}} \end{aligned}$$

Note that the elasticity $\varepsilon _{1}$ remains at its pre-merger level as demand is isoelastic.

Slutsky symmetry implies $\partial X_{j}/\partial P_{i}=\partial X_{i}/\partial P_{j}$ for $i,j=1,2$. From the definition of cross-price elasticities, we know that $\partial X_{j}/\partial P_{i}=\varepsilon _{ij}X_{j}/P_{i}$ where $\varepsilon _{ij}$ denotes the (constant) cross-price elasticity from *i* to *j*. Using these two equations, we therefore have:

11 $$\begin{aligned} \frac{X_{2}}{X_{1}}=\frac{\varepsilon _{21}}{\varepsilon _{12}}\frac{P_{1}}{ P_{2}} \end{aligned}$$

Moreover,

$$\begin{aligned} D_{12}=\frac{\varepsilon _{12}}{\varepsilon _{1}}\frac{X_{2}}{X_{1}}=\frac{ \varepsilon _{21}}{\varepsilon _{1}}\frac{P_{1}}{P_{2}} \end{aligned}$$

where the first equality follows from the definition of diversion ratios and the second equality follows from (11). Since $\varepsilon _{1}$ and $\varepsilon _{21}$ are constants, we thus have:

$$\begin{aligned} D_{12}^{*}=D_{12}\frac{P_{2}}{P_{1}}\frac{P_{1}^{*}}{P_{2}^{*}} \end{aligned}$$

Substituting this into (10), using $\left( P_{1}-C_{1}\right) /P_{1}=1/\varepsilon _{1}$ from the pre-merger first-order conditions and rearranging, we find:

12 $$\begin{aligned} \frac{P_{1}^{*}-C_{1}}{P_{1}^{*}}=\frac{P_{1}-C_{1}}{P_{1}}+D_{12} \frac{P_{2}^{*}-C_{2}}{P_{2}^{*}}\frac{P_{2}}{P_{1}} \end{aligned}$$

(and similarly for firm 2).

Solving this system of equations for $P_{1}^{*}$, using the fact that $P_{i}/C_{i}=1/\left( 1-M_{i}\right)$ and rearranging, we then find expression (7) in the main text. As in Shapiro’s (1996) symmetric case, (7) is well-defined only if diversion ratios are sufficiently small so $\Delta P_{1}/P_{1}$ remains non-negative (else an infinite price increase would ensue).

## Footnotes

1 U.S. Department of Justice & Federal Trade Commission, Horizontal Merger Guidelines (2010).

2 E.g., see *Somerfield plc & Wm Morrison Supermarkets plc* (Competition Commission final report of 5 September 2005); *LOVEFiLM International Limited/Amazon Inc. DVD rental subscription business* (OFT decision of 15 April 2008).

3 European Commission, Guidelines on the assessment of horizontal mergers under the Council Regulation on the control of concentrations between undertakings (OJ 2004 C31/5, 5 February 2004), at para. 28 and 29.

4 Case T-5/02, *Tetra Laval v. Commission*, 25.10.2002, (2002) II ECR 4381; Case T-310/01, *Schneider v. Commission*, 22.10. 2002, (2002) II ECR 4071; Case T-342/99, *Airtours v. Commission, *11.10.2001, (2002) ECR II 2585.

5 Council Regulation (EC) 139/2004 (OJ 2004 L24/1, 29.1.2004). See Neven (2006) for a historical perspective.

6 See Sect. 2 below for case references.

7 See Sect. 3 below for case references.

8 Since many (if not most) of the relevant cases predate our time at the Commission, this is necessarily a highly personal view. For recent surveys, see Buettner (2016), Buettner et al. (2016) and the annual agency updates in this *Review*.

9 Around the time of the completion of this article, the European General Court overturned the Commission’s prohibition decision *Hutchison 3G UK/Telefónica UK* in Case No. T–399/16, *CK Telecoms UK Investments v. Commission* (General Court judgement of 28 May 2020). As will be discussed in Sect. 2.4 below, this judgment concerns, inter alia, the standard of proof in European merger control. An assessment of its legal implications is beyond the scope of this paper, however, which instead focuses on the application of economic methodologies.

10 See Shapiro (2010) for a discussion.

11 E.g., see Case M.8744 *Daimler/BMW/Car Sharing JV* (Commission decision of 7 November 2018); Case M.9693 *Novelis/Aleris * (Commission decision of 1 October 2019); Case M.9739 *FCA/PSA* (Commission decision of 21 December 2020).

12 Here and in what follows, we display formulas for firm 1 only. The respective expressions for firm 2 can be found by exchanging subscripts 1 and 2.

13 Note that market delineation will typically exclude many products from the relevant market. If a direct comparison between implied and actual shares is desired—e.g., to assess whether firms offer closer substitutes than market shares suggest—it is therefore necessary to focus on diversion within the proposed market (however defined). More generally, it is sensible to exclude diversion to non-consumption, since abstinence would otherwise be attributed a “market share.”

14 See Buettner (2016) for a discussion of this argument in the context of recent EU cases.

15 Equivalently, raising price can be viewed as a positive externality vis-à-vis the merger partner, who will recapture some of the lost sales following a price increase.

16 Here, and elsewhere, we assume that prices are strategic complements.

17 We express CMCRs as a percentage of price rather than cost here, so as to facilitate the comparison with other measures of price pressure.

18 The (local) equivalence between the pass-on of marginal cost efficiencies and anti-competitive effects is explored in Froeb et al. (2005). It implies that it would be inconsistent to argue that marginal cost reductions cannot outweigh anti-competitive effects merely because pass-on is low. After all, the same low pass-through would also limit the pass-on of price pressure into post-merger prices. Of course, the potential for such an inconsistency applies in both directions (see footnote 53 below for an example).

19 The incremental value is assumed to be identical across consumers.

20 See Neurohr (2019) for a formal derivation.

21 See Jaffe and Weyl (2013) for a formal analysis.

22 For this reason, Neurohr’s (2019) description of CMCRs as “full upward pricing pressure” is somewhat of a misnomer, as CMCRs generically differ from the pre-merger tax that would generate post-merger prices.

23 Case M.6497 *Hutchison 3G Austria/Orange Austria* (Commission decision of 12 December 2012); Case M.6992 *Hutchison 3G UK/Telefónica Ireland* (Commission decision of 28 May 2014); Case M.7018 *Telefónica Deutschland/E-plus* (Commission decision of 2 July 2014); Case M.7419 *TeliaSonera/Telenor/JV* (withdrawn, 11 September 2015); Case M.7612 *Hutchison 3G UK/Telefónica UK* (Commission decision of 11 May 2016); Case M.7758 *Hutchison 3G Italy/Wind/JV* (Commission decision of 1 September 2016); Case M.8792 *T-Mobile NL/Tele 2 NL* (Commission decision of 27 November 2018).

24 As noted in the introduction, the UK mobile decision was recently overturned in court in *CK Telecoms v. Commission*, supra note 9.

25 See Buettner (2016) for a discussion.

26 We explore merger simulation and its relation to price pressure tools in Sections 2.3 and 2.4 below.

27 *Hutchison 3G Austria/Orange Austria*, supra note 23; Case M.8851 *BASF/Bayer Divestment Business* (Commission decision of 30 April 2018); Case M.9730 *FCA/PSA*, supra note 11.

28 Case M.5658 *Unilever/Sara Lee* (Commission decision of 17 November 2010); Case M.7421 *Orange/Jazztel* (Commission decision of 19 May 2015).

29 European Commission, supra note 3, at para. 28. Recall from (3), however, that these are cumulative (not alternative) factors as far as static effects are concerned.

30 E.g., see* Zipcar/Streetcar* (Competition Commission final report of 22 December 2010); *Unilever/Alberto Culver Co* (OFT decision of 16 June 2011); *Shell UK Ltd/Consortium Rontec Investments LLP* (OFT decision of 3 February 2012); *Saint-Gobain/Build Center* (OFT decision of 8 February 2012); *Edmundson Electrical Limited/Electric Center* (OFT decision of 11 May 2012); *Rexel UK Limited/Certain Assets of Wilts Wholesale Electrical Company Limited* (OFT decision of 23 January 2013); *Cineworld Group plc/City Screen Limited* (CMA final report of 8 October 2013); *The Original Bowling Company Ltd/Bowlplex Ltd* (CMA decision of 15 September 2015); *J Sainsbury PLC/Asda Group Ltd* (CMA final report of 25 April 2019).

31 CMA, Retail mergers commentary (10 April 2017), at para. 5.14. Many other NCAs also started using price pressure tools following the publication of the Guidelines. For example, Baltzopoulos et al. (2015) survey merger cases of the Swedish NCA that involved price pressure analyses.

32 This is not self-evident, since the transition between pre- and post-merger prices is not a marginal, but a discrete change. Hence, pass-through rates and diversion ratios may change along this transition.

33 Indeed, they tend to be challenging even in the most advantageous academic settings (e.g., see Miller et al. 2017a).

34 See Crooke et al. 1999 for calibration results.

35 E.g., see *Hutchison 3G UK/Telefónica Ireland*, supra note 23; *Telefónica Deutschland/E-plus*, supra note 23; * TeliaSonera/Telenor/JV*, supra note 23; *Orange/Jazztel*, supra note 28; *Hutchison 3G UK/Telefónica UK*, supra note 23; * Hutchison 3G Italy/Wind/JV*, supra note 23; *T-Mobile NL/Tele 2 NL*, supra note 23; Case M.8864 *Vodafone/Certain Liberty Global Assets* (Commission decision of 18 July 2019).

36 E.g., see Crooke et al. 1999.

37 E.g., see Hausman (2003) at 27 (noting that concave demand “*while theoretically possible, would not be expected for most new products and services*”).

38 Without Slutsky symmetry, the IPR formula is bulkier (see Hausman et al. 2011) as there can be multi-product interactions between the pass-through rates of products 1 and 2 in that case.

39 For cases using linear IPRs, see, for instance, *LOVEFiLM/Amazon*, supra note 2; *Sports Direct/JJB Sports* (Competition Commission final report of 16 March 2010).

40 The only information that is required in addition to diversion ratios and margins are market shares.

41 This difference can be meaningful for average market prices. The change in the merging firms’ own prices tends to be more moderate, since a price reaction to a price reaction is a second order effect.

42 See also Werden (1998), Crooke et al. (1999), Miller et al. (2017b) and CMA, supra note 31 at 34 (which states that the CMA will use linear or isoelastic demand to compute IPRs).

43 See, for instance, *Somerfield/Morrison*, supra note 2; * Co-operative Group Limited/Somerfield Limited* (OFT decision of 20 October 2008); *Co-operative Group Limited/Lothian Borders & Angus Co-operative Society Limited* (OFT decision of 6 March 2009); *WM Morrison Supermarkets plc/Co-operative Group Limited* (OFT decision of 10 July 2009); *Asda Stores Limited/Netto Foodstores Limited* (OFT decision of 23 September 2010); *Asda/Co-operative Group (5 stores)* (CMA decision of 28 November 2014).

44 E.g., see *Somerfield/Morrison*, supra note 2 at 7.26.

45 See Werden and Froeb (2008) for a survey.

46 E.g., see the discussion in Angrist and Pischke (2010), Nevo and Whinston (2010), and Knittel and Metaxoglou (2011).

47 Case M.4854 *TomTom/Tele Atlas* (Commission decision of 14 May 2008); Case M.5644 *Kraft Foods/Cadbury* (Commission decision of 6 January 2010); *Unilever/Sara Lee*, supra note 28; *Telefónica Deutschland/E-Plus*, supra note 23; *TeliaSonera/Telenor*, supra note 23.

48 Note that the other terms are simply the Lerner equations from the pre-merger profit-maximization.

49 We will return to this topic in Sect. 4 when we discuss the role of market shares.

50 We obtain a similar result analytically in Annex 6.2, which ranks different price pressure tools based on varying pre-merger pass-through rates.

51 As we show in Annex 6.2, this range is typically a narrower subset of the range that is provided by calibrated merger simulation (as described in Sect. 2.3 above).

52 *Hutchison 3G Italy/Wind/JV*, supra note 23, Annex A at para. 10.

53 For an exception, see *Telefónica/E-plus*, supra note 23, Annex A at para. 140 (defendants arguing that demand could in theory also be concave). As was noted in footnote 18, however, a low pass-through rate would have equally affected the merging parties’ efficiency claims. As was shown in Eq. (4), the question whether marginal cost efficiencies outweigh anticompetitive effects is independent of the exact rate of pass-through.

54 E.g., see *Hutchison 3G Italy/Wind/JV*, supra note 23, at para. 774 and Annex A at para. 48. A frequently contested point is the degree of diversion to outside goods. See Moresi and Zenger (2018) for a formal treatment.

55 *CK Telecoms v. Commission* supra note 9; *Hutchison 3G UK/Telefónica UK*, supra note 23.

56 The Commission has appealed the judgment in front of the European Court of Justice.

57 E.g., see the recent prohibition decision in *Sainsbury/Asda*, supra note 30.

58 CMA, supra note 31, at para. 5.14.

59 As the Guidelines point out, potential mitigating factors—such as entry or product repositioning—have to be timely, likely and sufficient to overcome competition concerns (§6.1). See Caradonna et al. (2020) for a recent model that explores the limits of such countervailing factors.

60 See *Hutchison 3G/O2 Ireland*, supra note 23, at para. 315, 578, 582; *Telefónica/E-plus*, supra note 23, at para. 493; * Orange/Jazztel*, supra note 28, at para. 334 and 360; *Hutchison 3G Italy/Wind/JV*, supra note 23, Section 7.2.

61 See Case M.7932 *Dow/DuPont* (Commission decision of 27 March 2017); Case M.7962 *ChemChina/Syngenta* (Commission decision of 5 April 2017); Case M.8084 *Bayer/Monsanto* (Commission decision of 21 March 2018).

62 See Federico et al. (2019) and Kokkoris and Valletti (2020) for more general recent treatments.

63 See Shapiro (2011) for an early critique of this literature with regard to its implications for merger enforcement.

64 For instance, when considering changes in the intensity of competition, the theoretical framework of Aghion et al. (2005) varies some uniform conduct parameter that is common to all firms. Similarly, Aghion et al. (2001) proxy competition by a symmetric change in an industrywide parameter of product differentiation.

65 The paper is particularly specific to mobile telephony mergers. In this context, Motta and Tarantino show that when efficiencies can also be achieved by contractual solutions such as network sharing, a merger could nonetheless be challenged, since less restrictive contractual solutions would be available to reach the efficiency gains without reducing price competition.

66 See Gilbert (2019) for a related model.

67 This sequential setting where investment is chosen first, followed by price competition, arises from the long-term nature of investment decisions.

68 As noted by the Guidelines, “*The Agencies evaluate the extent to which successful innovation by one merging firm is likely to take sales from the other, and the extent to which post-merger incentives for future innovation will be lower than those that would prevail in the absence of the merger*” (§6.4).

69 This notwithstanding, consumers are typically worse-off after a merger because, for a given level of innovation, prices will increase as a result of less product market competition.

70 A positive price coordination effect is assumed at the outset in Federico et al. (2017).

71 The relevance of asymmetric outcomes on R&D effort has long been discussed in the literature on R&D joint ventures starting with D’Aspremont and Jacquemin (1988). There is a debate as to when interior versus corner equilibria apply in practice (see, e.g., Leahy and Neary 2005). This discussion has sharpened our understanding but certainly not diminished the importance and policy impact of the seminal work of d’Aspremont and Jacquemin.

72 See European Commission, supra note 3 at 8, 38.

73 See Federico et al. (2019) for a survey.

74 E.g., see Case M.7275 *Novartis/GSK (Oncology Business)* (Commission decision of 28 January 2015); *Dow/DuPont*, supra note 61; * Bayer/Monsanto*, supra note 61; Case M.9294 *BMS/Celgene* (Commission decision of 29 July 2019).

75 E.g., see Case M.7278 *General Electric/Alstom* (Commission decision of 8 September 2015); Case M.7477 *Halliburton/Baker Hughes* (withdrawn, 2 May 2016).

76 E.g., see Case M.6166 *Deutsche Börse/NYSE Euronext* (Commission decision of 1 February 2012).

77 This part is based on Buehler et al. (2017) where more details can be found.

78 The essential component of a formulated crop protection product is the active ingredient (AI), which is the result of innovation efforts that are made by R&D-oriented agrochemical companies over a period of approximately 10–11 years.

79 For one merging party, the patent analysis revealed that, while having the smallest patent portfolio, its patents were on average of a significantly higher quality than those of its competitors. Controlling for patent quality, it was therefore either the number-one or number-two innovator in crop protection overall, depending on the concrete measure that was used for patent quality.

80 E.g., see Kaplow (2010) or Ginsburg and Wright (2015).

81 See Farrell and Shapiro (2010b) and the references cited therein.

82 As Larry White (the editor) correctly points out, finding a significant GUPPI for two merging products technically implies that those products by themselves constitute a relevant market according to the hypothetical monopolist test. Any further efforts at market definition—either before or after a GUPPI determination—may therefore add little in many cases.

83 E.g., if your last beverage yesterday evening was a glass of wine and your first beverage this morning was a cappuccino, then this does not imply “competitive switching” between wine and coffee. See Chen and Schwartz (2016) and Conlon and Mortimer (2020) for analyses of such potential biases in diversion data.

84 A similar point can be made with respect to margins, whose construction is often contested – in particular as regards which costs are truly incremental.

85 See Hovenkamp and Shapiro (2018) for a discussion.

86 E.g., see Nocke and Schutz (2018), Nocke and Whinston (2020) or Spiegel (2020).

87 At the time of writing of this article, the COVID-19 pandemic is still recent and ongoing. It remains unclear what structural impact the pandemic will have on different industries. We observe that the financial crisis of 2007-2008 did not halt or reverse the long-term trend towards increasing market power.

88 *Secretary of State for Home Department v. Rehman*, [2002] 3 WLR 8TT, at 895.

89 See Valletti and Zenger (2018, 2019) for a discussion.

90 See Ashenfelter et al. (2014) and the studies cited therein for systematic evidence that anti-competitive mergers often do not involve the creation of a dominant position.

91 For a discussion, see the empirical literature on merger simulation retrospectives (e.g., Björnerstedt and Verboven 2016, and the references cited therein).

92 *D* will be strictly constant with linear demand, or logit demand, or in the absence of an outside good, for instance. The simulation results of Miller et al. (2016) suggest that also for other standard demand forms, the change in diversion ratios from pre- to post-merger equilibrium will often be modest.

93 $\rho$ is the rate at which a cost increase that solely affects firms 1 and 2 is passed through into final prices. Since mergers cause a discrete change in price, $\rho$ is not the point-estimate of pass-through at *P*, but the average pass-through rate that is measured over the discrete change from *P* to $P^{*}$.

94 $\rho ^{*}$ is the rate at which a cost increase that solely affects the merged entity is passed through into final prices. It denotes the average pass-through rate that is measured over the discrete change from $P^{*}$ to *P*.
